# Mediating roles of activities of daily living and depression on the relationship between sleep quality and health-related quality of life

**DOI:** 10.1038/s41598-024-65095-0

**Published:** 2024-06-18

**Authors:** Xiao-Qing Ren, Gong-Ming Zhao, Shuo-Wen Fang, Ling-Feng Xu, Li-Dan Wang, Lin-Hai Zhao, Man-Man Lu

**Affiliations:** 1 School of Health Management, Anhui Medical University, No.81, Meishan Road, Shushan District, Hefei, 230032 Anhui China; 2 School of Public Health, Fudan University, Shanghai, 200032 China; 3 Health Policy Research Center, Anhui Medical University, Hefei, 230032 Anhui China

**Keywords:** Sleep quality, ADL, Depression, HRQOL, Loneliness, Moderated mediation model, Depression, Quality of life

## Abstract

This study aimed to explore the mediating effects of ADL and depression on the relationship between sleep quality and HRQOL among older people in rural China, while also exploring the moderating impact of loneliness. The study gathered data from a household survey conducted among 1587 Chinese rural older adults (mean age = 73.63 years). The collected data was analyzed using SPSS version 23.0 software (IBM, New York, USA) and the PROCESS macro version 4.0 program. The findings indicated a significant correlation between sleep quality, ADL, depression, loneliness and HRQOL. ADL and depression exhibited a chain mediation effect on the relationship between sleep quality and HRQOL. Notably, the association between sleep quality and HRQOL was entirely mediated by ADL and depression. Additionally, loneliness acted as a moderator in the relationship between ADL and HRQOL. The findings of this study suggest that interventions focusing on sleep quality should prioritize strategies for enhancing older adults’ ADL and depression as integral components of promoting older adults’ HRQOL.

## Introduction

The advancements in healthcare have led to a substantial increase in the global population of older adults, posing significant challenges on a global scale. Projections indicate that the percentage of individuals aged 65 and above will escalate from 10% in 2022 to 16% by 2050^1^. In China, the issue of aging is particularly salient, as the older people surpassed 200 million by the close of 2021, constituting 14.2% of the total populace^2^, with expectations of further growth. Rural aging in China is progressing notably faster than in urban areas, influenced by factors like economic development and population migration^3^. However, prior research indicates that the health-related quality of life (HRQOL) of rural older adults tends to be lower than their urban counterparts^4,5^. HRQOL, a subjective and multidimensional measure of physical and mental health status along with social functioning, is commonly utilized to evaluate the well-being of the older adults, reflecting emotional, physical, and social aspects^6,7^. The World Health Organization stresses that the primary aim of healthy aging is to enhance the quality of life for the older adults, thereby extending healthy life expectancy. Thus, prioritizing the HRQOL of older adults in rural areas is crucial for the effective implementation of China’s healthy aging policy.

Older adults commonly face the health challenge of declining sleep quality. Sleep quality is a complex concept encompassing sleep efficiency, sleep latency, sleep duration, and wake after sleep onset^8^. Clinical studies demonstrate that advancing age often brings challenges in falling and staying asleep, resulting in fragmented sleep patterns^9^. A substantial proportion of older adults globally, estimated between 40 and 60%, face inadequate sleep quality^10^. Moreover, sleep issues can contribute to conditions like hypertension, diabetes, and obesity^11,12^, as well as restrict activities of daily living (ADL)^13^, consequently impacting the HRQOL of older adults. Previous studies have established the link between sleep quality and HRQOL^14,15^, with emerging evidence suggesting that depression may be a significant factor in this relationship^16^. A study centered on older Chinese adults revealed a connection between sleep quality and symptoms of depression^17^. Nonetheless, further elucidation is needed to understand the interplay among sleep quality, ADL, depression, and HRQOL.

Loneliness, a subjective and distressing negative emotion, frequently coexists with and closely relates to depression^18^. Clinical studies reveal a high prevalence of loneliness among older adults displaying depressive symptoms^19^. Longitudinal research indicates that loneliness can forecast depressive symptoms in the older adults, a correlation that persists even after adjusting for confounding factors^20^. Furthermore, a reciprocal relationship between loneliness and sleep quality is acknowledged. Studies suggest that inadequate sleep quality in China and Japan may heighten feelings of loneliness among older adults^21,22^. Conversely, a meta-analysis revealed that loneliness in older adults can heighten the risk of diminished sleep quality^23^. What is more, research on the link between ADL and loneliness in older adults also shows a significant association. ADL impairment has been associated with heightened levels of loneliness^24,25^, while loneliness has been linked to increased risks of ADL decline^26^. Therefore, loneliness may act as a moderating factor in a specific pathway between sleep quality and HRQOL.

Despite extensive exploration of the relationship between sleep quality and HRQOL, the underlying mechanisms of this association largely remain obscure. Specifically, uncertainties persist regarding the mediating role of ADL and depression in this relationship, as well as the potential moderating impact of loneliness. Poor sleep quality can lead to diverse adverse outcomes, such as physical fatigue, diminished concentration, and memory impairments, impeding daily functioning. These consequences may affect emotions, prompt the progression of depression, and subsequently influence an individual’s overall quality of life and well-being. Understanding the intricate interplay among these factors is imperative. By elucidating the potential mechanisms through which sleep quality affects HRQOL, valuable insights and recommendations can be offered to enhance the quality of life for the older adults, thereby positively contributing to the health and happiness of rural older adults.

**Figure 1:**
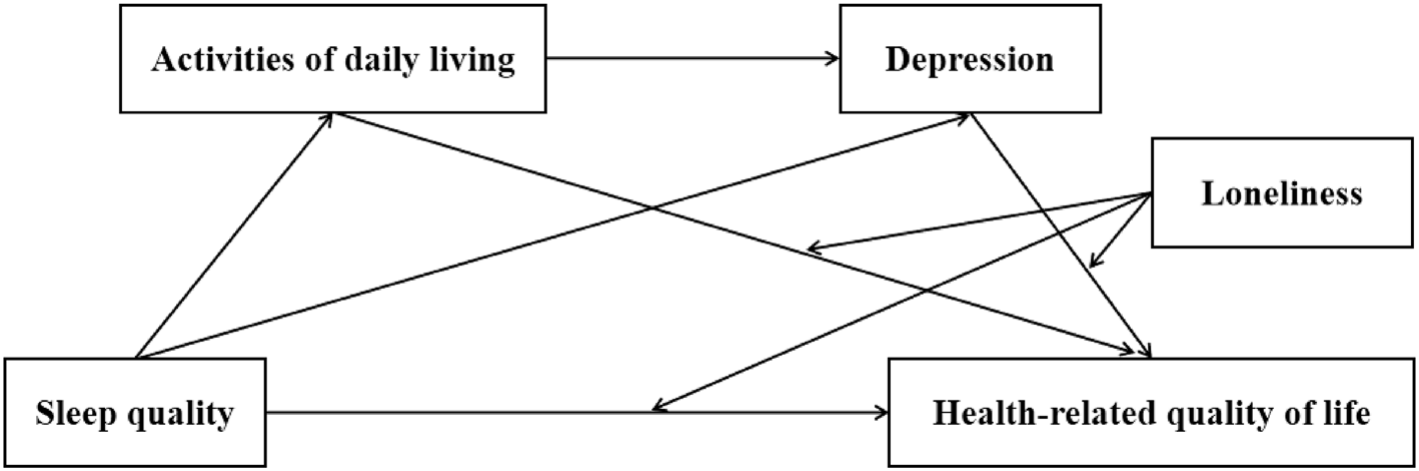
Theoretical model: a moderated mediation model.

Hence, the present study aimed to explore the mediating effects of ADL and depression on the relationship between sleep quality and HRQOL among older people in rural China, while also exploring the moderating impact of loneliness. Drawing from previous research, we have formulated a theoretical model (Fig. 1) and put forth the following hypotheses: (1) Sleep quality is positive related to HRQOL. (2) ADL mediates the relationship between sleep quality and HRQOL. (3) Depression mediates the relationship between sleep quality and HRQOL. (4) ADL and depression play a chain mediation role between sleep quality and HRQOL. (5) Loneliness plays a moderating role in the relationships mentioned above.

## Methods

### Study design and participants

This study was a cross-sectional survey that utilized a self-report questionnaire. The survey was carried out between June 2021 to August 2021 in rural areas located in Anhui Province, China. All methods were performed in accordance with the Declaration of Helsinki-Ethical Principles for Medical Research Involving Human Subjects and other relevant laws, regulations and ethical norms. To ensure the feasibility and sample representativeness, a multi-stage, whole-cluster random sampling method was utilized. Firstly, Anhui Province was stratified into three distinct regions: North Anhui, Central Anhui, and South Anhui, based on its geographical and economic characteristics. Subsequently, two districts/counties were randomly selected from each region, for a total of six districts/counties. Secondly, a total of 18 townships were chosen by selecting three from each of the districts/counties sampled. Thirdly, 72 villages were randomly selected from the sampled townships, with four villages from each. Finally, individuals who met the inclusion criteria from the selected villages were invited to participate in the study. The study received approval from the Biomedical Ethics Committee of Anhui Medical University (Approval No. 2020H011).

The inclusion criteria were as follows: (1) being aged 60 years and above; (2) effective communication ability; (3) informed consent and willingness to cooperate; and (4) residence in a rural area. The exclusion criteria: Individuals who self-reported their inability to complete the questionnaire due to a medical diagnosis of a major illness by an institution at the county level or higher, including conditions like stroke, severe cognitive impairment, and mental disorders. The sample size was calculated using the following formula: n = [z^2^ × p × (1-p)]/d^2^. The confidence interval (CI) was set at 95% (z-value = 1.96). The ADL loss rate (p) among older adults was found to be 23.80%^27^, and the acceptable error margin (d) was determined to be 0.1p, which calculated a sample size of n = 1230. Considering a 20% non-response rate and sample loss, the total estimated sample size was calculated to be 1538. The surveyors, who had received standardized training, briefed the participants on the survey’s objectives and guaranteed the confidentiality of their personal information. After obtaining informed consent, the questionnaire was conducted via in-person visits to households. As a token of appreciation, all participants who completed the questionnaire were given a small gift. In total, 1622 participants completed the questionnaires. After evaluation, there were 1587 qualified questionnaires, with a qualified rate of 97.84%.

### Measures and instruments

#### Basic information questionnaire

An original questionnaire was used to gather demographic data including gender, age, marital status, education, personal annual income, chronic diseases, alcohol consumption, smoking, and regular exercise (Appendix 1).

#### Pittsburgh sleep quality index scale (PSQI)

The PSQI Scale developed by Buysse et al. was used to measure sleep quality during the previous month^28^. The PSQI has been widely used and is a reliable indicator that can reflect global sleep status in different populations, including general populations and individuals with sleep problems or mental disorders, over the past month. The study employed a total of 18 questions to assess sleep quality across 7 dimensions: subjective sleep quality, sleep latency, sleep duration, habitual sleep efficiency, sleep disturbances, use of sleeping medication and daytime dysfunction. The PSQI score was calculated by summing the scores for each dimension, which range from 0 to 3 points. Therefore, the PSQI score can range from 0 to 21 points. A higher PSQI score indicates worse sleep quality. In analysis of sleep quality characteristics and related factors in the older adults in rural China, the sensitivity and specificity of the Chinese version of the PSQI were found to be 98.3% and 90.2%, respectively^29^. In this study, the Cronbach’s α for this scale was 0.670.

#### The Barthel Index

The Barthel Index is a tool for clinical evaluation of ADL frequently employed in various cultural contexts^30^. In the present study, the 10-item scale was used, which included the following parts: feeding, bathing, grooming, dressing, bowel control, bladder control, toilet use, transfers, mobility, and stair climbing. The scale comprises two items scoring 0 or 5 points, six items scoring 0, 5, or 10 points, and the last two items scoring 0, 5, 10, or 15 points^31^. The score ranges from 0 (total dependence) to 100 (complete independence), the higher the value, the better the functional status. It was generally believed that a full score of 100 indicates that the patient’s daily living ability was normal and does not need to rely on; 61 ~ 99 points indicated that patients could basically take care of themselves, which was mild dependence; 41 ~ 60 was divided into moderate dependence, ≤ 40 was divided into severe dependence, patients need help and life dependence was obvious. The structural validity and reliability of the Chinese version of the Barthel Index were demonstrated to be strong among Chinese older adults^32^. In this study, the Cronbach’s α for this scale was 0.831.

#### Patient health questionnaire-9 (PHQ-9)

Depressive symptoms were assessed by the PHQ-9, a screening tool widely applied to detect and measure depression and severity in clinical settings in the last two weeks^33^. The response options were measured with a four-point Likert scale (0 = not at all, 1 = several days, 2 = more than half the days, and 3 = nearly every day), with higher scores indicating higher levels of depressive symptoms. The total score ranges from 0 to 27, with a score of 5 or higher indicating the presence of depressive symptoms^34^. The PHQ-9 has demonstrated strong reliability and validity among older Chinese adults^35^. In this study, the Cronbach’s α for this scale was 0.812.

#### Three-item loneliness scale

The concept of loneliness was measured utilizing three items drawn from the UCLA Loneliness Scale, which was developed by Hughes and colleagues^36^. The study of Liu et al. in Chinese older adults confirmed that the scale had good reliability and validity^37^. The scale was used to measure the loneliness of the older adults over the past week. “I feel left out”, “I feel isolated” and “I lack companionship” were measured from three questions. The three-point score of “1 (hardly ever) ~ 3 (often)” was used. The total score on the scale ranges from 3 to 9, with a higher score indicating a higher level of loneliness. In this study, the Cronbach’s α for this scale was 0.880.

#### Three-level EuroQol five-dimensional health questionnaire (EQ-5D-3L)

This study employed the EQ-5D-3L to assess HRQOL among rural older adults. The EQ-5D-3L comprises five dimensions: mobility, self-care, usual activities, pain/discomfort, and anxiety/depression, and each dimension has three severity levels (no, moderate, and severe problems) defining a total of 243 health states^38^. The EQ-5D-3L descriptive system can convert each health state into a utility score using a country-specific value set based on social preferences^39^. The utility score varied from 0 (representing death) to 1 (representing perfect health). A preference weight set for the Chinese population was applied to estimate the mean EQ-5D utility score, which has been demonstrated to have good reliability and validity^40^. In this study, the Cronbach’s α for this scale was 0.604.

### Statistical analysis

SPSS version 23.0 software (IBM, New York, USA) and the PROCESS macro version 4.0 program were used for the data analysis. Descriptive analysis was conducted on the socio-demographic and clinical characteristics of the participants. Pearson correlation analysis was employed to examine the relationships between sleep quality, ADL, depression, loneliness, and HRQOL. A multiple mediation analysis was performed to establish a model involving these variables: an independent variable, a dependent variable, and two mediator variables. Within this model, sleep quality and HRQOL were determined to be the independent variable and the dependent variable, respectively. ADL and depression established pathways from sleep quality to HRQOL. The SPSS PROCESS macro (model 6) was employed to estimate the total, direct, and indirect effects^41^. The total effect (c) represented the relationship between sleep quality and HRQOL without controlling for ADL and depression. The direct effect (c′) reflected this relationship after controlling for ADL and depression, whereas the indirect effects denoted the impact of sleep quality on HRQOL via ADL, depression, or both. The bootstrap method, involving 5000 resampling iterations to establish robustness and accuracy, was utilized to establish 95% confidence interval (CI) for determining the significance of mediating effects. Significance was attributed to direct or indirect effects when the CI did not encompass zero. Furthermore, model 89 of PROCESS was used to test the moderating effect of loneliness. Gender, age, marital status, education, personal annual income, chronic diseases, drinking, smoking and regular exercise were included as control variables in the models.

### Ethics approval and consent to participate

The study was approved by the Biomedical Ethics Committee of Anhui Medical University (NO.2020H011). Participants gave informed consent to participate in the study before taking part.

## Results

### Common method deviation test

Since the measurement of all variables in this study comes from the self-report of the participants, to avoid the common method bias resulting, the Harman single factor test was employed^42^. The results showed that six factors with characteristic roots greater than 1 were obtained after factor analysis, and the first factor explained 18.06% of the variance, which was less than the critical value of 40%. Hence, the study’s outcomes were unaffected by common method bias.

### General characteristics of the participants

A total of 1587 participants were included in the study with the age of 60 years old and above (Mean = 73.63, SD = 6.45). Among these participants, 17.77% were over the age of 80 and 52.49% were female. 71.33% of the participants were married and only 10.90% had a junior high school education or higher. 73.34% of the participants had a personal annual income of less than 6,500 CNY. Only 21.11% had no chronic diseases, while 40.08% had one chronic disease and 38.81% had two or more. In addition, the data showed that 14.43% of participants were current smokers, 27.47% were current drinkers, and 37.49% had regular exercise. The demographic characteristics of all participants are summarized in Table 1.

**Table 1:** The demographic characteristics of participants (N = 1587).

Characteristics	N (%)
Gender	
Male	754 (47.51%)
Female	833 (52.49%)
Age	
60 ~ 69	450 (28.36%)
70 ~ 79	855 (53.88%)
≥ 80	282 (17.77%)
Marital status	
Married	1132 (71.33%)
Other cases	455 (28.67%)
Education	
Pre-primary and below	901 (56.77%)
Primary school	513 (32.33%)
Junior high school or higher	173 (10.90%)
Personal annual income	
< 6500 CNY	1164 (73.34%)
6500–15,000 CNY	311 (19.60%)
> 15,000 CNY	112 (7.06%)
Chronic disease	
0	335 (21.11%)
1	636 (40.08%)
≥ 2	616 (38.81%)
Drinking	
Never	848 (53.43%)
Past	303 (19.09%)
Current	436 (27.47%)
Smoking	
Never	1005 (63.33%)
Past	353 (22.24%)
Current	229 (14.43%)
Regular exercise	
Yes	595 (37.49%)
No	992 (62.51%)

*CNY* Chinese Yuan.

### Descriptive statistics and correlation analysis

The results indicated that sleep quality was significantly and negatively associated with ADL (r = − 0.135, *P* < 0.01) and HRQOL (r = − 0.216, *P* < 0.01), and significantly and positively associated with depression (r = 0.520, *P* < 0.01) and loneliness (r = 0.219, *P* < 0.01). ADL was significantly and negatively associated with depression (r = − 0.305, *P* < 0.01) and loneliness (r = − 0.120, *P* < 0.01), and significantly and positively associated with HRQOL (r = 0.849, *P* < 0.01). Depression was significantly and negatively associated with HRQOL (r = − 0.432, *P* < 0.01), and significantly and positively associated with loneliness (r = 0.398, *P* < 0.01). In addition, there was a negative and significant association between loneliness and HRQOL (r = − 0.257, *P* < 0.01). More details are presented in Table 2.

**Table 2:** Descriptive analysis and bivariate correlations among variables.

Variables	Mean	SD	Sleep quality	ADL	Depression	Loneliness	HRQOL
Sleep quality	6.51	3.62	1				
ADL	95.46	10.74	− 0.135**	1			
Depression	4.57	4.71	0.520**	− 0.305**	1		
Loneliness	3.83	1.56	0.219**	− 0.120**	0.398**	1	
HRQOL	0.95	0.09	− 0.216**	0.849**	− 0.432**	− 0.257**	1

N = 1587; *ADL* Activities of daily living, *HRQOL* Health-related quality of life. **P < 0.01.

### Mediating effect test

After controlled variables such as gender, age, marital status, education, personal annual income, chronic diseases, drinking, smoking and regular exercise among rural older adults, a mediation effect test procedure was employed to assess the indirect impact of sleep quality on HRQOL. This indirect effect was found to be mediated by ADL and depression. The model’s fit and the significance of each path coefficient were evaluated using the PROCESS macro program in SPSS, as outlined by Hayes^41^.

**Figure 2:**
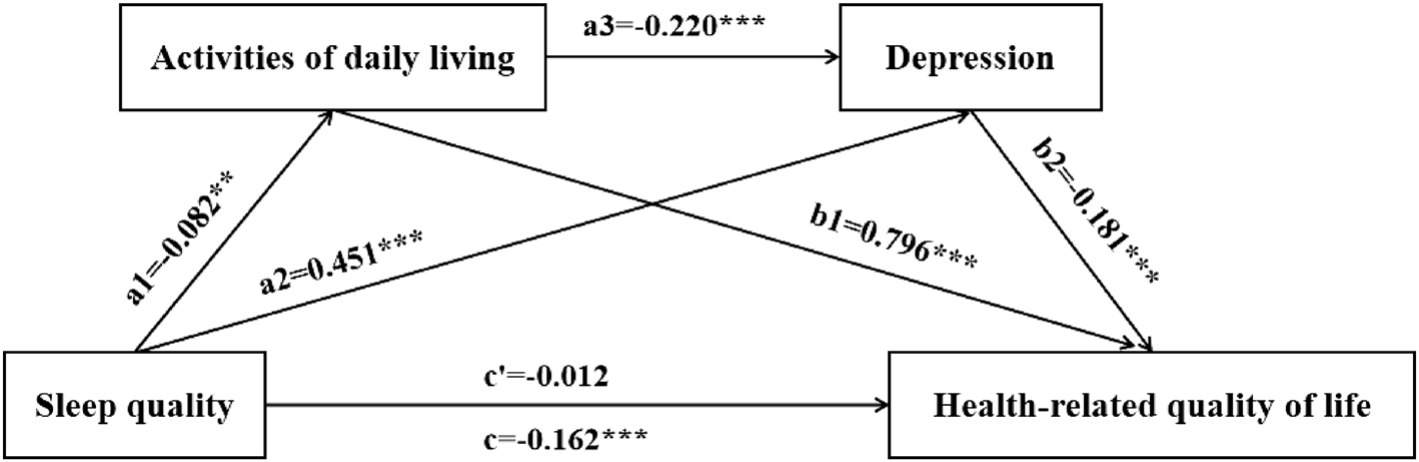
Chain-mediation pathway diagram (**P < 0.01, ***P < 0.001).

The results showed that sleep quality was significantly and negatively associated with ADL (*β* = − 0.082, *t* = − 3.249, *P* < 0.01), and significantly and positively associated with depression (*β* = 0.451, *t* = 21.206, *P* < 0.001). ADL was significantly and negatively associated with depression (*β* = − 0.220, *t* = − 10.367, *P* < 0.001), significantly and positively associated with HRQOL (*β* = 0.796, *t* = 59.321, *P* < 0.001). Depression was significantly and positively associated with HRQOL (*β* = − 0.181, *t* = − 11.703, *P* < 0.001). In addition, sleep quality total effect significantly negatively predicted HRQOL (*β* = − 0.162, *t* = − 6.464, *P* < 0.001). There was no significant direct association between sleep quality and HRQOL. More details are presented in Table 3 and Fig. 2.

**Table 3:** Regression model of the effect of Sleep quality on HRQOL.

Variables	ADL			Depression			HRQOL			HRQOL		
	β	SE	t	β	SE	t	β	SE	t	β	SE	t
Sleep quality	− 0.082	0.025	− 3.249**	0.451	0.021	21.206***	− 0.012	0.015	− 0.805	− 0.162	0.025	− 6.464***
ADL				− 0.220	0.021	− 10.367***	0.796	0.013	59.321***			
Depression							− 0.181	0.015	− 11.703***			
R	0.284			0.591			0.870			0.306		
R^2^	0.081			0.350			0.756			0.094		
F	13.817***			77.016***			407.094***			16.284***		

N = 1587; *ADL* Activities of daily living; HRQOL, Health-related quality of life. **P < 0.01, ***P < 0.001. Adjusted gender, age, marital status, education, personal annual income, chronic diseases, drinking, smoking and regular exercise.

All three indirect paths were significant. The mediating effect (the total indirect effect) was − 0.150, included path 1: Sleep quality → ADL → HRQOL (− 0.065); path 2: Sleep quality → Depression → HRQOL (− 0.082), and path 3: Sleep quality → ADL → Depression → HRQOL (− 0.003). The relative effect of paths 1, 2, and 3 were 40.30%, 50.37%, and 1.98%, respectively. Therefore, ADL and depression play a complete mediating role between sleep quality and HRQOL. The mediating effects, direct effect, and corresponding effect scales are shown in Table 4.

**Table 4:** Multiple mediated path analysis.

	Effect	SE	Bootstrap 95% CI		Effect ratio
			Lower	Upper	
Direct effect	− 0.012	0.015	− 0.041	0.017	7.35%
Indirect effect					
Path 1: sleep quality → ADL → HRQOL	− 0.065	0.023	− 0.111	− 0.021	40.30%
Path 2: sleep quality → depression → HRQOL	− 0.082	0.012	− 0.104	− 0.060	50.37%
Path 3: sleep quality → ADL → depression → HRQOL	− 0.003	0.001	− 0.006	− 0.001	1.98%
Total indirect effect	− 0.150	0.026	− 0.201	− 0.100	92.65%
Total effect	− 0.162	0.025	− 0.211	− 0.113	100%

N = 1587; *ADL* Activities of daily living, *HRQOL* Health-related quality of life. Adjusted gender, age, marital status, education, personal annual income, chronic diseases, drinking, smoking and regular exercise.

### Moderated chain mediating effect analysis

Table 2, illustrates significant correlations between loneliness and sleep quality, ADL, depression, and HRQOL. To investigate the potential moderating role of loneliness in the relationship between sleep quality and HRQOL, we conducted additional analyses to assess the path influenced by loneliness.

**Figure 3:**
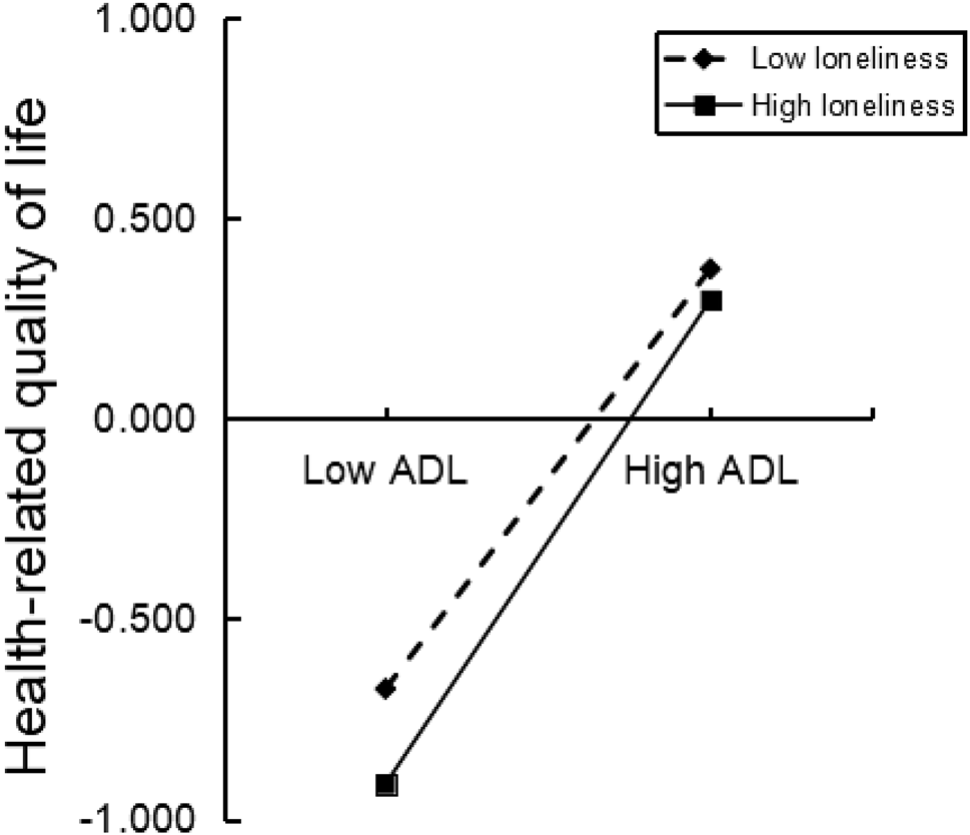
The moderating effects of loneliness on the relationship between ADL and HRQOL.

After standardizing the variables, PROCESS model 89 was used to test the moderating effect of loneliness, adjusted for covariables and the results are shown in Table 5. In this adjustment model, the effect value of ADL on HRQOL reached 0.775 (95% CI 0.749 ~ 0.802), excluding zero; the effect value of loneliness on HRQOL reached − 0.082 (95% CI − 0.111 ~ − 0.053), excluding zero; the interaction effect value between the two variables was 0.074 (95% CI 0.051 ~ 0.096), excluding zero. The 95% CI for the interaction between sleep quality and loneliness, as well as depression and loneliness, encompassed zero, suggesting that only the interaction between ADL and loneliness had a significant positive impact on HRQOL, as illustrated in Fig. 3. As shown in Fig. 3, when loneliness levels were low, ADL significantly predicts a positive effect on HRQOL (*Bsimple* = 0.736, *P* < 0.001); Conversely, at high levels of loneliness, the positive predictive effect of ADL on HRQOL was strengthened (*Bsimple* = 0.848, *P* < 0.001).

**Table 5:** Moderating effect test.

Variables	β	SE	t	Bootstrap 95% CI	
				Lower	Upper
Loneliness	− 0.082	0.015	− 5.497***	− 0.111	− 0.053
Sleep quality	− 0.013	0.014	− 0.896	− 0.041	0.015
Sleep quality × loneliness	− 0.010	0.014	− 0.736	− 0.036	0.017
ADL	0.775	0.014	57.569***	0.749	0.802
ADL × loneliness	0.074	0.011	6.446***	0.051	0.096
Depression	− 0.135	0.016	− 8.259***	− 0.168	− 0.103
Depression × loneliness	− 0.017	0.011	− 1.438	− 0.040	0.006
R^2^	0.774				
F	336.191***				

N = 1587; *ADL* Activities of daily living. **P < 0.01, ***P < 0.001. Adjusted gender, age, marital status, education, personal annual income, chronic diseases, drinking, smoking and regular exercise.

The significance of the moderated chain mediating effect was judged by detecting the significance of the difference between indirect effects, as shown in Table 6. When loneliness levels were low, defined as one standard deviation below the mean (Mean − 1 SD), the chain mediating effect was − 0.059 (95% CI − 0.101 ~ − 0.018), indicating a significant effect. when loneliness levels were high, defined as one standard deviation above the mean (Mean + 1 SD), the chain mediating effect was − 0.068 (95% CI − 0.120 ~ − 0.021), also demonstrating a significant effect. The difference in the chain mediating effect between these two situations was − 0.009 (95% CI − 0.021 ~ − 0.001), excluding zero. Therefore, it can be inferred that loneliness acts as a moderator on the chain mediating.

**Table 6:** Moderated chain mediation effect values and confidence intervals.

Path	Moderating variable	Mediation effect	SE	Bootstrap 95% CI	
				Lower	Upper
Sleep quality → ADL → HRQOL	Low loneliness	− 0.059	0.021	− 0.101	− 0.018
	High loneliness	− 0.068	0.025	− 0.120	− 0.021
	Difference	− 0.009	0.005	− 0.021	− 0.001

N = 1587; *ADL* Activities of daily living, *HRQOL* Health-related quality of life. Adjusted gender, age, marital status, education, personal annual income, chronic diseases, drinking, smoking and regular exercise.

## Discussion

This study aims to explore the underlying mechanism by which sleep quality influences HRQOL among older adults in rural China. Using a chain-mediated model, the research findings reveal a significant negative association between poor sleep quality and HRQOL. Specifically, the analysis demonstrates that ADL and depression act as mediators in the link between sleep quality and HRQOL, functioning in a chain-mediated manner. Furthermore, it is observed that loneliness plays a moderating role in the relationship between ADL and HRQOL. Thus, the hypotheses H1 to H5 introduced in this study are essentially validated.

This study shows that the overall impact of sleep quality, as measured by the PSQI index, is inversely associated with HRQOL, aligning with prior studies^7,43,44^. This suggests that a decrease in sleep quality could result in a decline in HRQOL. Notably, this study revealed that the link between sleep quality and HRQOL lost its significance once the two mediating variables of ADL and depression were introduced. This pattern indicated that ADL and depression jointly mediated the connection between sleep quality and HRQOL. The outcomes indicated that sleep quality initially predicted HRQOL without accounting for mediating variables. However, when ADL and depression were considered as mediating variables, their presence mediated the relationship between sleep quality and HRQOL. This indicates that sleep quality is a more distal factor influencing HRQOL, with sleep behavior related to personal plans and awareness, while depression and disability were more direct factors affecting HRQOL. The sleep disturbance model suggests that sleep disorders adversely affect physical health by weakening emotional regulation and disrupting the body’s circadian rhythm^45^. Hence, the impact of sleep quality on HRQOL is primarily mediated through an indirect pathway.

This study explores the potential mediating roles of ADL and depression in the relationship between sleep quality and HRQOL among older adults in rural areas. The results initially indicate that decreased sleep quality correlates with deterioration in ADL performance, which subsequently leads to reduced levels of HRQOL. Existing research has shown that inadequate sleep duration adversely affects cognitive function and physical abilities^46,47^. Cognitive effects include memory decline and difficulty concentrating, impacting daily tasks, while physical effects involve reduced endurance, strength, and flexibility for activities like transferring or climbing stairs. These consequences ultimately contribute to a decline in older adults’ quality of life. Furthermore, the study suggests that sleep quality influences HRQOL through its association with depression. Clinical evidence suggests that chronic insomnia may increase the risk of depression by disrupting the balance of the hypothalamic–pituitary–adrenal axis^48^. Previous studies have also demonstrated a significant correlation between depression and decreased quality of life^49,50^. For instance, the China Hainan Centenarian Cohort Study found that depression negatively influenced HRQOL in older age^51^. This may be attributed to poor sleep quality often coinciding with reduced cognitive and physical function, known precursors to depression^44,52,53^. These findings underscore the importance of maintaining high-quality sleep for enhancing physical and mental well-being among older adults in rural communities. In light of these findings, healthcare providers should prioritize enhancing ADL in rural older adults and evaluating their mental health status, particularly in individuals with inferior sleep quality, to preempt possible deterioration in HRQOL.

In addition to examining the individual mediating roles of ADL and depression, this study delved into the chain mediation effect between sleep quality and HRQOL. The findings of this study revealed that the deterioration of sleep quality was initially associated with a decline in ADL performance, followed by the accumulation of depressive symptoms, ultimately leading to a decrease in HRQOL. The correlation between sleep quality and ADL aligns with existing research. Previous studies have also demonstrated that impaired independence in ADL among older adults may increase their vulnerability to developing depressive symptoms^16,54–56^. Older adults with diminished capacity to perform daily activities were restricted in their engagement with the environment, limiting social interactions and information exchange, which can culminate in the accumulation of negative emotions and the onset of depression^57,58^. Moreover, a high level of depression is likely to result in lower HRQOL scores^59^. Therefore, enhancing the quality of life among rural older adults necessitates addressing depression and physical ailments beyond solely addressing sleep disturbances.

This study found that loneliness moderated the latter portion of Path 1 (Sleep quality → ADL → HRQOL). Specifically, the influence of ADL on HRQOL was more notable among rural older adults with higher levels of loneliness compared to those with lower levels. A longitudinal study indicated that loneliness was associated with increased challenges in daily activities^60^. The mechanisms linking these variables were not fully understood, but potential pathways may involve heightened inflammation, altered immune function, and changes in health behaviors^61^. These pathways were believed to be driven by loneliness, leading to physical impairments related to ADL and consequently declining HRQOL. To enhance HRQOL in older rural Chinese adults, priority should be given to early efforts to improve sleep quality, mental health, and ADL levels.

Based on the study findings, it is recommended that government departments take the following actions: (1) Implement health education programs focusing on rural community sleep health to disseminate knowledge of good sleep practices among older adults, raise awareness of healthy sleep habits, and encourage the adoption of a healthy lifestyle. (2) Establish an integrated healthcare and retirement system in rural areas, enhance training in activities of daily living, provide rehabilitation nursing and exercise guidance, offer a diverse range of cultural and recreational activities to improve the daily living skills of the older adults, bolster social interactions and support networks, and alleviate feelings of loneliness and depression. Additionally, healthcare providers are advised to: (1) Set up sleep monitoring stations in rural communities to provide monitoring services, deliver personalized guidance on sleep to older adults, and reduce the risk of sleep disorders. (2) Develop a comprehensive mental health service system for rural communities, conduct mental health educational initiatives for the older adults, create a helpline for mental health consultations, offer psychological counseling and support services, facilitate interventions and treatments for older adults with psychological issues, and enhance the mental well-being and social support of older adults. These initiatives demand collaborative efforts from government bodies, society, and families to enhance the well-being of aging rural populations.

While the study has offered valuable insights, it is crucial to acknowledge its limitations. The cross-sectional design provides valuable associative data but lacks temporal considerations, limiting the ability to draw causal inferences from the findings. Additionally, the self-reported data collection approach could introduce reporting biases. Furthermore, the study focused solely on older adults residing in rural areas, without conducting a parallel study in urban settings for comparison. Future research should expand to urban areas and incorporate longitudinal methods to gain a comprehensive understanding of the intricate relationships among sleep quality, ADL, depression, loneliness, and HRQOL in rural older adults.

## Conclusion

Overall, the sleep quality of older adults not only directly predicts their quality of life but also impacts it through the chain-mediated effects of depression and ADL. Furthermore, the indirect association between ADL and HRQOL is moderated by feelings of loneliness. These findings enhance our understanding of how sleep quality affects the quality of life in rural older adults, offering insights into enhancing the well-being of older rural individuals with sleep problems and guiding targeted interventions to boost their overall welfare.

### Supplementary Information

Supplementary Information 1.

Supplementary Information 2.

Supplementary Information 3.

## Data Availability

The data presented in this article are available. The content related to the privacy of participants is not publicly available. The datasets used and/or analyzed during the current study available from the corresponding author on reasonable request.

## Abbreviations

ADL: Activities of daily living
HRQOL: Health-related quality of life
PSQI: Pittsburgh sleep quality index
PHQ: Patient health questionnaire
EQ-5D-3L: Three-level EuroQol five-dimensional health questionnaire
