# Supplementary material for: Mediating roles of activities of daily living and depression on the relationship between sleep quality and health-related quality of life
Source: Sci Rep. 2024 Jun 18;14:14057. doi: 10.1038/s41598-024-65095-0 (PMC11189409; doi:10.1038/s41598-024-65095-0)
Supplement: Supplementary file 1 — Supplementary Information 1. [file 41598_2024_65095_MOESM1_ESM.docx]

**Appendix 1. Original questionnaire used to gather demographic and clinical information.**

1.1 Name

1.2 Date of birth Year Month

1.3 Gender.

Ⅰ Female; Ⅱ Male

1.4 Education.

Ⅰ Pre-primary and below; Ⅱ Primary school; Ⅲ Junior high school or higher

1.5 Marital status.

Ⅰ Married. a. First marriage, marriage year; b. Remarriage, from the most recent marriage, to date year; Ⅱ Never married; Ⅲ Divorced year; Ⅳ Widowed, not married again, widowed years; Ⅴ Separation; Ⅵ Other______

1.6 What is your annual income (not including that of your family members)?

Ⅰ <6500 CNY; Ⅱ 6500-15000 CNY; Ⅲ 15000-24000 CNY; Ⅳ 24000-75000 CNY; Ⅴ >75000 CNY

1.7 Please indicate whether you suffer from any of the following chronic diseases, which have been diagnosed by a medical institution at the county level or above (Multiple choices allowed).

Ⅰ Type 1 or 2 diabetes; Ⅱ Hypertensive; Ⅲ Hyperlipidemia; Ⅳ Chronic hepatitis;

Ⅴ Malignant tumor, specific part Ⅵ Heart attack;

Ⅶ Chronic lung diseases (tuberculosis, chronic obstructive pulmonary disease, etc.);

Ⅷ Mental illness, type Ⅸ Stroke; Ⅹ Other

1.8 Alcohol drinking in the past 1 year.

Ⅰ Never; Ⅱ Current; Ⅲ Past

1.9 Smoking.

Ⅰ Never; Ⅱ Current; Ⅲ Past

1.10 Do you engage in daily exercise consistently?

Ⅰ No; Ⅱ Yes, exercise minutes
